# Behavioral and pathophysiological outcomes associated with caffeine consumption and repetitive mild traumatic brain injury (RmTBI) in adolescent rats

**DOI:** 10.1371/journal.pone.0187218

**Published:** 2017-11-06

**Authors:** Glenn R. Yamakawa, Connor Lengkeek, Sabrina Salberg, Simon C. Spanswick, Richelle Mychasiuk

**Affiliations:** University of Calgary, Department of Psychology, Calgary, Alberta, Canada; University of Florida, UNITED STATES

## Abstract

Given that caffeine consumption is exponentially rising in adolescents and they are at increased risk for repetitive mild traumatic brain injury (RmTBI), we sought to examine the pathophysiological outcomes associated with early life caffeine consumption and RmTBI. Adolescent male and female Sprague Dawley rats received either caffeine in the drinking water or normal water and were then randomly assigned to 3 mild injuries using our lateral impact device or 3 sham procedures. Following injury induction, behavioral outcomes were measured with a test battery designed to examine symptoms consistent with clinical manifestation of PCS (balance and motor coordination, anxiety, short-term working memory, and depressive-like behaviours). In addition, pathophysiological outcomes were examined with histological measures of volume and cellular proliferation in the dentate gyrus, as well as microglia activation in the ventromedial hypothalamus. Finally, modifications to expression of 12 genes (*Adora2a*, *App*, *Aqp4*, *Bdnf*, *Bmal1*, *Clock*, *Cry*, *Gfap*, *Orx1*, *Orx2*, *Per*, *Tau*), in the prefrontal cortex, hippocampus, and/or the hypothalamus were assessed. We found that chronic caffeine consumption in adolescence altered normal developmental trajectories, as well as recovery from RmTBI. Of particular importance, many of the outcomes exhibited sex-dependent responses whereby the sex of the animal modified response to caffeine, RmTBI, and the combination of the two. These results suggest that caffeine consumption in adolescents at high risk for RmTBI should be monitored.

## Introduction

Concussion, or mild traumatic brain injury (mTBI) is the most common type of head injury and is often sustained during sports or recreation [1]. mTBI differs from moderate or severe TBI in that no gross structural damage can be identified when using conventional brain imaging techniques. Adolescents and children are at the highest risk for mTBI and repetitive mTBI (RmTBI), which increases their risk of sustaining subsequent concussions, extends recovery time, and may lead to the development of long-term psychological and/or neurological deficits [2–4]. Longitudinal measurements of patients that have experienced RmTBI showed poor executive function, depression scores, and cognitive changes that were related to the number of injuries received [5]. In addition, resting state fMRI indicates sustained brain network hyper-connectivity in high school football players when compared to non-contact sport controls [6]. Even in the absence of a concussion, neuropsychological impairment was linked to the number and location of head blows that they received throughout the season [7]. Head impact telemetry systems have shown that high school football players may experience as many as 1000 impacts throughout the season [8]. Therefore, a thorough understanding of RmTBI pathology, particularly during the developmentally vulnerable stage of adolescence is critical to predicting outcomes.

Moreover, we currently lack prognostic tools that can effectively distinguish between those that will recover without incident and those that will go on to suffer from lingering symptomology commonly referred to as post-concussive syndrome (PCS). Premorbid characteristics such as diet and cognitive reserve have been shown to modify recovery from mTBI and contribute to increased heterogeneity in adolescent cohorts [9, 10]. Children and adolescents are the fastest growing population of caffeine users, with caffeine use in adolescents rising exponentially [11]. This exponential increase has been associated with increased accessibility and the emergence of caffeinated soda and energy drinks. Caffeine is socially acceptable, legal for children and adolescents, and is the most widely used psychoactive substance in the world. Adolescents, (10–16 year of age) who report caffeine use, consumed at least 2 servings per day [12]. Of significance, adolescent awareness of the potential negative consequences associated with daily consumption of caffeine is low, while primary reasons for consumption included staying awake and having extra energy for extracurricular activities including sports [13]. Caffeine has been shown to have beneficial effects for adults in the context of sports whereby it may increase voluntary muscle activation and vertical jump height [14, 15]. This is possibly why some research has found that in populations of adolescent athletes, caffeinated energy drinks were consumed by 69% of participants, with 17% using them every day or more than 3 times a week [16].

Given that caffeine consumption and RmTBI are common in adolescents, especially in the context of sports, we sought to examine the pathophysiological outcomes associated with early life caffeine consumption and RmTBI. Both male and female rats received either caffeinated or non-caffeinated drinking water and were then randomly assigned to 3 mild injuries using our lateral impact device or a sham procedure. Following injury induction, behavioral outcomes were measured with a behavioral test battery designed to examine symptoms consistent with clinical manifestation of PCS. In addition, pathophysiological outcomes were examined with histological processing for volume, microglia activation, and cellular proliferation, along with modifications to gene expression in the prefrontal cortex (PFC), the hippocampus (HPC) and the hypothalamus (HYPO). The PFC and HPC were chosen for analysis because mTBIs and caffeine alter executive function, short-term working memory, impulsivity, and attentional processes, all of which rely on neural circuits associated with these 2 brain regions. The HYPO plays a significant role in regulation of sleep-wake cycles, metabolism, and hormone secretion, functions also possibly influenced by caffeine and brain injury.

## Materials and methods

### Animals and RmTBI procedure

Male (n = 35) and female (n = 39) Sprague Dawley rats were bred in-house to 7 dams (Charles Rivers Laboratories, QC, Canada). All rats were housed in same-sex groups of 3 or 4 and were maintained on a 12:12 hr light:dark cycle in a temperature controlled husbandry room at 21°C. Food and water (caffeinated or non-caffeinated) was available *ad libitum*. Rats were randomly assigned to the caffeine (1g/L in water; Sigma Aldrich, Oakville ON) or non-caffeinated group and were then further assigned to the RmTBI or sham condition. This generated the following groups; caffeine + RmTBI (11M: 13F), caffeine + sham (8M: 9F), water + RmTBI (9M: 9F), and water + sham (7M: 8F). We used a dose of caffeine (1g/L) that has routinely been used in the literature to induce serum levels roughly equivalent to 5 cups of coffee/day [17, 18]. The experiments and procedures were approved by the University of Calgary Conjoint Facilities Research Ethics Board, and conducted in accordance with the Canadian Council of Animal Care.

RmTBIs were administered using our lateral impact device as described previously [19, 20]. The RmTBI and sham groups were subjected to three sham or injury procedures spaced three days apart (P30, P34 & P38). The rats were anesthetized using inhalant isoflurane gas until they were no longer responsive to a toe pinch (~30 s). Next, they were placed in the prone position on a Teflon® board with the left side of the head facing the lateral impactor device. A 50-g cylindrical weight was propelled towards the head at 7.40 m/s ± 0.64 using a pneumatic air compressed barrel. The weight made impact with a small aluminum plate placed against the rat’s head. The purpose of this plate was to reduce the risk of bone or skull damage while still ensuring rotational, acceleration, and deceleration forces were imposed. Following the procedure, Xylocaine (2%; AstraZeneca, Canada) was applied to head and rats were placed on their backs in a clean warm cage. For sham injuries, the rats were anesthetized, placed on the Teflon® board, but not impacted. The *time-to-right*, determined as the time taken for the rat to right itself from the position on its back with no muscle tone, to a prone position with muscle tone, was recorded.

### Behavioral testing

**Fig 1:**
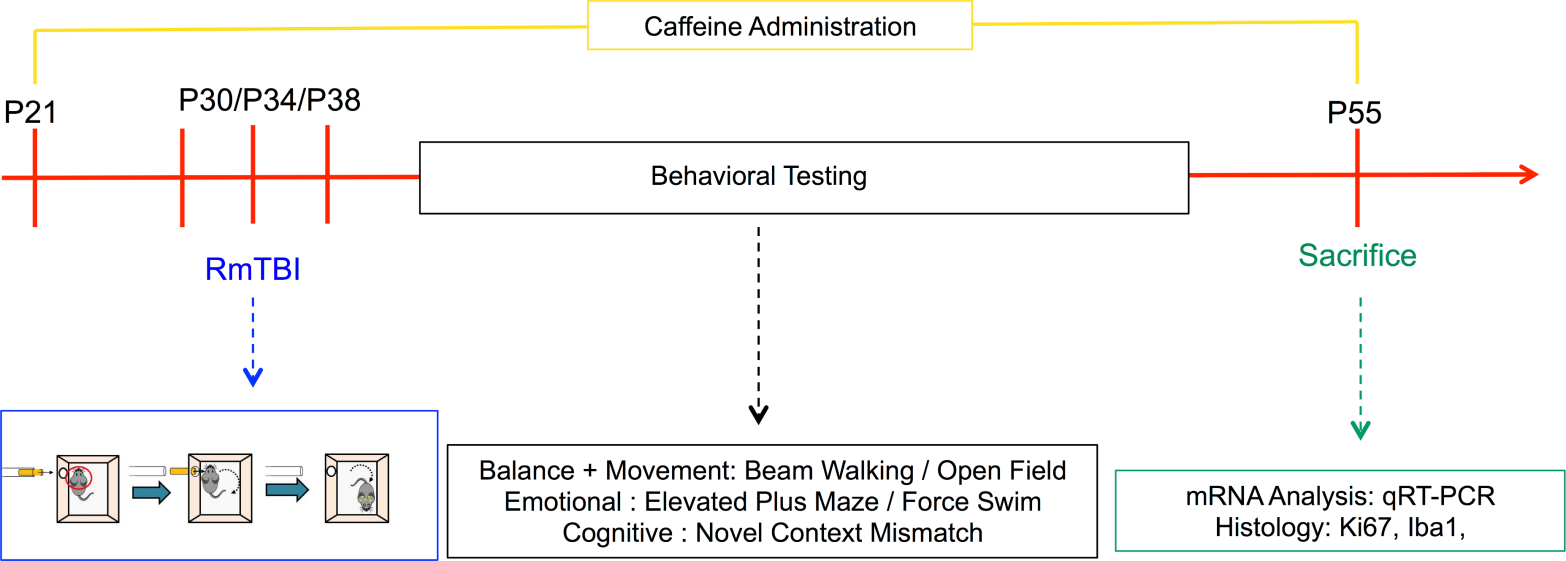
Illustrative representation of the experimental timeline.

These behavioral tests were chosen to model the symptoms associated with post-concussive syndrome as has been previously discussed [21, 22] The beam-walk test was undertaken first 24 hours following mTBI to measure loss of balance and coordination. The open field and elevated plus maze were undertaken next as motor abnormalities and anxiety type behaviors emerge next. Next, the novel context was conducted as typically working memory deficits occur around this time. Finally, the forced swim was conducted last as this procedure is typically seen as the most stressful and also resembles the time when depressive behaviors emerge. See Fig 1 for a illustration of the experimental paradigm.

#### Beam-Walking

Twenty-four hours following the third mTBI, (post-injury day 1 (PID1)) all rats were tested using the beam walking procedure [23]. This procedure was designed to assess balance and motor coordination impairments that are often seen following concussion. Rats were placed at one end of a 165 cm long tapered beam with their home cage on the other end. The start of the beam was wider and as the beam approached the home cage it becomes narrower. The beam was suspended between two platforms approximately 1 m off the ground and had 2 cm ledges that catch the hind legs if the rat slipped off the central portion of the beam. Each rat underwent 1 unscored pre-training trial. The following 4 trials were videotaped and scored by an observer blind to the experimental condition. Hind leg foot slips were scored every time the rat used the safety ledge with the rear foot while moving across the beam. The beam was cleaned with Virkon® between each rat.

#### Open-Field

On PID2, rats were tested in the open field procedure. The rats were placed in the centre of an open field arena 135 cm in diameter that was monitored by an overhead video camera. The rat was permitted to freely move about for 10 min undisturbed. The open field test has been used to assess general locomotor activity and the capacity to engage in exploratory behavior [24]. The distance and speed of travel was obtained with the overhead video camera. The open field was cleaned using Virkon® between each rat.

#### Elevated plus maze (EPM)

On PID3, rats were tested using the elevated plus maze (EPM). The EPM was constructed of black plexiglass® and had two open arms and two closed arms. This behavioral task has been used extensively to examine anxiety-like behaviors [25]. Rats were placed in the centre of the EPM, facing one of the closed arms and were permitted to explore for 5 min. The session was recorded with a video camera and a researcher blinded to the experimental conditions scored each session for the time the rat spent in the open and closed arms. The EPM was cleaned using Virkon® between each rat.

#### Novel context mismatch (NCM*)*

The NCM has been used a measure of short-term working memory [26]. On PID5-7, the rats were placed in two different contexts (Context A and B) for 5 min each, one context immediately preceding the other. Context A was a clear plastic rectangular bin containing two identical objects. Context B was a dark blue circular bin containing a different pair of identical objects. On PID8 rats were exposed to a probe trial; Context A (5 min) → Context B (5 min) → home-cage (5 min) → Novel Context (5 min). The novel context consisted of a modified Context A (Context A^*modified*^ contained one object from context A and one object from context B) or a modified Context B (Context B^*modified*^ which contained one object from context B and one object from context A). Exploration of the novel context was videotaped, and a research associate blinded to the experimental conditions recorded the amount of time each rat spent investigating the novel object and the old object. All of the objects and context containers were cleaned with Virkon® between each testing session.

#### Forced swim

The forced swim procedure has been used extensively in many different situations to measure depressive type behaviors [27]. Animals that spend more time immobile during the procedure are thought to display increased depressive-like behaviors. A cylindrical tank 30 cm diameter 60 cm high was filled with warm water (~25°C). At PID 9 or 10, the rat was gently placed in the water in the cylinder for the testing session and recorded for 7 min. Upon completion of the testing session, the rat was dried with a warm towel and returned to the home cage. A research analyst blinded to the experimental conditions scored the amount of time each rat spent immobile (absence of swimming movement).

### Molecular analysis

Upon completion of the behavioral testing (~ P55), 1/2 of the rats were sacrificed for RT-qPCR whereby brain tissue was collected from the PFC, HPC, and HYPO. Rats were anesthetized using isoflurane, weighed, and decapitated rapidly. The PFC, HPC, and HYPO were extracted using the coordinate system provided by the Paxinos and Watson (2006) rat atlas and flash frozen on dry ice. The brain tissue was stored at -80°C until RNA was extracted.

#### RNA extraction and RT-qpcr

The RNA was extracted using the Allprep RNA/DNA Mini Kit (Qiagen, Hilden, Germany) following the protocols of the manufacturer. A Nanodrop2000 (Thermo Fisher Scientific, Waltham, MA) was used to determine the total RNA concentration. 2-μgs of the purified RNA was reverse transcribed to cDNA using oligo(dT)_20_ of the Superscript III First-Strand Synthesis Supermix kit (Invitrogen, Carlsbad, CA) following the manufacturer’s protocols. The RT-qPCR reaction contained 10 ng cDNA combined with 0.5 μM of the forward and reverse primers and SYBR Green FastMix and was analyzed with a CFX Connect Real-Time PCR Detection System (Bio-Rad, Hercules, CA). To determine the PCR efficiency, a standard curve was generated using serial dilution of cDNA from pooled control samples. Each sample was run in duplicate along with no-template controls for each gene that was examined in each brain region. Gene expression in each of the experimental conditions was determined by normalization to 2 housekeeping genes (*CycA* and *Ywhaz*) [28] using the previously validated 2 ^-ΔΔCt^ method [29].

### Serum caffeine concentration

At the time of sacrifice, trunk blood was collected from all animals in serum separator tubes (BD, Franklin Labs, NJ, USA). Samples were clotted for 30 minutes at room temperature and then centrifuged at 1000g for 15 minutes. Serum was aliquoted into 300–400 μl samples and stored at -20°C. ELISA kits were purchased for caffeine (Abraxis LLC, PA, USA) and were performed according to the manufacturer’s instructions. All standards, positive and negative controls, and samples were run in triplicate, and measured with the BioTek Synergy H.T. plate reader and Gen5 2.00.18 software using a path length correction algorithm. All samples fell within normal range of the standard curve.

### Gene selection

A total of 12 genes were selected for analysis. In the PFC and HPC we examined gene expression changes in 6 genes, adenosine A_2A_ receptor (*Adora2a*), amyloid precursor protein (*App*), aquaporin-4 (*Aqp4*), brain derived neurotrophic factor (*Bdnf*), glial fibrillary acidic protein (*Gfap*), and Tau protein (*Tau*). Caffeine is an *Adora2a* antagonist, and it is believed that caffeine’s neuroprotective abilities are linked to suppression of *Adora2a* activation [30]. *App* expression has been demonstrated to increase following TBI [31], while caffeine has been shown to reduce levels of *App* in animal models of neurodegeneration [32]. *Aqp4* channels have been shown to play an important role in water transport and studies in TBI have demonstrated that increased A*qp4* reduces edema following TBI and is necessary for recovery [33]. *Bdnf* is frequently assessed following brain injury and has been linked to the neuroprotective properties of caffeine [34–36]. *Gfap* has been consistently used as an estimate of astrocyte activation following brain injury [37, 38]. *Tau* is a microtubule associated protein that has become well-established biomarker of axonal injury following mTBI [39, 40]. Eight genes were examined in the HYPO; 6 of which are involved in the typical transcriptional feedback loop of the circadian clock, brain muscle ARNT like protein 1 (*Bmal1*), circadian locomotor output cycles kaput (*Clock*), cryptochrome (*Cry*), period (*Per*), orexin receptor 1 (*Orx1*), and orexin receptor 2 (*Orx2*). Given that both RmTBI and caffeine consumption disrupt typical sleep patterns, we sought to determine if changes in circadian clock genes were involved [41, 42]. In addition to the circadian clock genes, we examined expression of *App* and *Tau* in the HYPO for the reasons described above.

### Histology

The remaining half of the rats were injected with an overdose of sodium pentobarbital and were intracardially perfused with approximately 200 ml of phosphate buffered saline (0.1M PBS) followed by a similar volume of 4% paraformaldehyde (PFA). Brains were extracted and stored in 4% PFA for 24 hours after which they were transferred to a 30% sucrose, 0.1M PBS solution. Upon sinking, brains were sectioned using a freezing sliding microtome. Sections were taken employing a sampling fraction of 1/12 and were cut at a thickness of 40 microns.

#### Volume analysis

To estimate the volume of the dentate gyrus granule cell layer (DG) a single series of tissue was stained with Cresyl Violet using standard laboratory procedures. Images of brain sections were captured using a Zeiss Axioplan 2 microscope attached to a Zeiss Axiocam 503 camera with a 5x/0.25 objective. Using ImageJ software (https://imagej.nih.gov/ij/) a sampling grid was randomly placed over each captured image of the DG. An area per point of 0.01 mm^2^ was used, and the total number of contact points between the granule cell layer and the grid was quantified for each section. The area associated with each point was then multiplied by the number of contact points, the section cut thickness, and the sampling fraction. These numbers were summed to provide a volume estimate of the dentate gyrus granule cell layer.

#### Immunohistochemistry

Free-floating sections were incubated in a primary solution containing either rabbit anti-Ki67 (Millipore), or rabbit anti-Iba1 (Wako Lab Chemicals) at a dilution of 1:500 in 0.1M PBS, 0.3% Triton-X for 24 hours. Sections were then incubated in a secondary solution containing a 1:500 dilution of anti-rabbit Cy3 (Jackson ImmunoResearch) for 24 hours. Tissue was counter-stained with 4,6-Diamidino-2-phenylindole (DAPI; Sigma Aldrich), and then mounted and coverslipped using a fluorescent mounting medium, and stored at 4°C until quantification.

#### Quantification

All quantification was performed using a Ziess Axioplan 2 microscope matched to a 40x/0.75 objective. The number of immune-positive cells within each designated region of interest (DG granule cell layer, ventromedial hypothalamus) were counted in each section, except for those in the uppermost focal plane in order to minimize edge artifacts (Kronenberg et al., 2006). The total number of immune-positive cells was multiplied by the inverse of the section-sampling fraction (1/12 for the DG and 3/12 for the HYPO) to provide a total number estimate.

### Statistical analysis

All data were analyzed using three-way ANOVAs with sex (male; female), caffeine (caffeine; non-caffeine) and injury (RmTBI; sham) as factors. Post hoc follow-up pairwise comparisons were conducted where applicable. All statistical analyses were conducted using SPSS 20.0 for Mac and considered significant if p < 0.05. For all graphs means are displayed ± standard error.

## Results

### Effects of caffeine on animal characteristics

**Fig 2:**
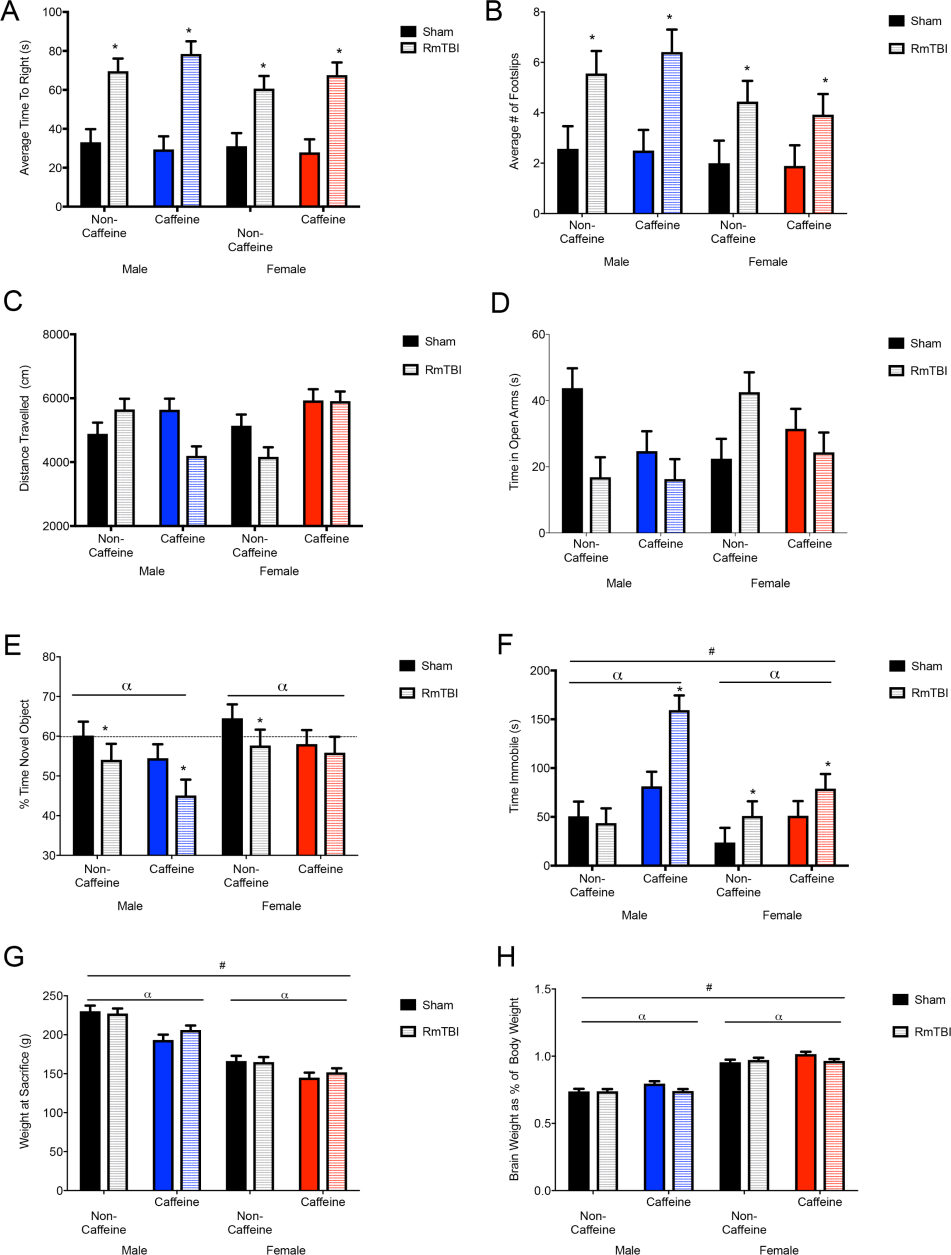
Behavioral test battery outcomes, and body and brain weights. Data are displayed as the means with the error bars depicting the standard error. Mean differences were considered significant at p< .05. (*) indicates an effect for RmTBI, (α) indicates an effect for caffeine, and (#) indicates a main effect of sex. (A) The average time to right in seconds is a measure of loss of consciousness and was significantly greater in male and female mTBI groups in both the non-caffeine and caffeine condition. (B) Rats receiving RmTBI showed a significantly greater number of rear foot slips when crossing the beam. (C) The distance travelled during the open field showed a sex by caffeine by injury interaction. Males in the non-caffeine condition showed an increase in distance traveled with RmTBI and a reduction in distance in the caffeine condition whereas the females in the non-caffeine condition showed a reduction in distance with mTBI and no change in the caffeine condition. (D) The time spent in open arms in the EPM showed sex by injury and a sex by caffeine by injury interactions with decreases in time seen with RmTBI. The exception was the female non-caffeinated RmTBI condition showing increased time in the open arms. (E) Displays the percent of time spent with novel object during the novel context mismatch. There were main effects for caffeine and injury. The horizontal dashed line indicates expected amount of time rats should spend with the novel object. (F) Time spent immobile during the forced swim test. There was a main effect for sex and caffeine in addition to an effect of injury. (G) The mean body weight for each group upon conclusion of the experiment is displayed. There were significant effects for sex and caffeine consumption between the groups, with females and caffeine groups having significantly lower body weights. (H) The brain weights as a percentage of body weight showed effects for sex and caffeine in addition to a RmTBI by caffeine interaction. Male and female rats receiving RmTBI and caffeine showed significantly lower brain weights when controlling for body weight.

Analysis of ELISA data demonstrated that animals in the caffeine group had significantly higher levels of caffeine in serum as compared to animals in the non-caffeine group (10.58 mg/L ± 0.23 vs. 0.11 mg/L ± .37, respectively). The three-way ANOVA exhibited a main effect of caffeine *F*(1, 73) = 344.60, *p* < .01. Similar to human reports, caffeine consumption reduced overall weight gain in our rats. At the time of sacrifice (~P50), caffeine-exposed rats weighed significantly less than controls. The three-way ANOVA exhibited a main effect of sex, *F*(1, 73) = 163.54, *p* < .01, and of caffeine, *F*(1, 73) = 26.75, *p* < .01. Finally, examination of brain weight (measured as a percentage of body weight) demonstrated that females had heavier brains than males, caffeine increased brain weight in control rats, but caffeine decreased brain weight in TBI rats. The three-way ANOVA demonstrated a main effect of sex, *F*(1, 73) = 338.49, *p* < .01, and of caffeine, *F*(1, 73) = 5.34, *p* = .02. There was a significant interaction between caffeine and injury, *F*(1, 73) = 6.61, *p* = .01 with reductions in relative brain weight seen in the caffeine groups following injury See Fig 2G. All other main effects and interactions were non-significant (*p* > 0.05).

### Behavioral analysis

Rats in the RmTBI group exhibited acute impairments in the time-to-right and beam-walking task, indicating that the injury induction protocol was effective (Fig 2A and 2B). In the open field, males in the non-caffeine condition exhibited an increase in distance traveled following RmTBI, but a reduction in distance if they were exposed to chronic caffeine. Females in the non-caffeine condition showed a reduction in distance following RmTBI, however no change in the caffeine condition (Fig 2C). The time spent in open arms in the EPM exhibited a significant sex by injury and three-way interaction whereby RmTBI had an effect in the non-caffeinated condition, but not the caffeine group, and caffeine rats significantly differed from non-caffeinated controls (Fig 2D). Short-term working memory was significantly affected by sex, injury, and caffeine (Fig 2E). Males in the caffeine condition showed larger deficits in working memory, whereas females in the caffeine condition appeared to have these deficits ameliorated. Finally, rats that experienced RmTBI or caffeine exposure, exhibited increased immobile time in the forced swim task (Fig 2F). Males receiving caffeine showed higher levels of immobility, with the highest amount of time seen in the male caffeine RmTBI group. See Table 1 for a summary of the three-way ANOVA results for the 6 behavioural tasks. All other effects and interactions were non-significant (*p* >0.05).

**Table 1:** Summary of statistical results from the three-way ANOVAs for the behavioral measures in question.

Behavioral Test	Effect of Sex: F(*p*)	Effect of Injury: F(*p*)	Effect of Caffeine: F(*p*)	Significant Interactions
Time-to-Right	1.48 (.23)	64.88 (< .01)	0.22 (.64)	N/A
Beam Walk	2.93 (.09)	16.56 (< .01)	0.03 (.96)	N/A
Open Field	0.55 (.46)	3.08 (.08)	2.56 (.14)	*Sex x Caffeine =* 9.62 (< .01) *Three-way Interaction =* 9.15 (< .01)
Elevated Plus Maze	3.7 (.31)	1.46 (.23)	2.40 (.13)	*Sex x Injury =* 6.78 (.01) *Three-way Interaction =* 6.07 (.01)
Novel Context Mismatch	3.78 (.05)	4.59 (.03)	4.02 (.04)	N/A
Forced Swim	8.09 (< .01)	7.60 (.01)	19.45 (< .01)	*Sex x Caffeine =* 3.95 (.05)

### Molecular analysis

See Table 2 for a summary of the three-way ANOVA results for the mRNA changes in the three different brain regions examined.

**Table 2:** Summary of the statistical results for the three-way ANOVAs for the 6 genes of interest in the PFC, HPC, and HYPO.

Brain Region	Gene	Effect of Sex:F(*p*)	Effect of Injury: F(*p*)	Effect of Caffeine: F(*p*)	Significant Interactions
	*Adora2a*	0.02 (.89)	2.13 (.16)	5.01 (.03)	N/A
**PFC**	*App*	4.58 (.04)	2.05 (.16)	0.06 (.81)	*Three-way interaction* = 6.24 (.02)
	*Aqp4*	3.39 (.07)	0.88 (.36)	8.94 (< .01)	N/A
	*Bdnf*	6.47 (.02)	7.12 (.01)	0.31(.58)	N/A
	*Gfap*	0.21 (.66)	5.80 (.02)	22.55 (< .01)	*Sex x Caffeine =* 5.19 (.03)
					*Injury x Caffeine =* 4.56 (.04)
	*Tau*	3.12 (.09)	4.32 (.05)	1.77 (.19)	N/A
	*Adora2a*	17.96 (< .01)	3.21 (.08)	0.14 (.72)	*Injury x Caffeine =* 7.66 (.01)
					*Three-way interaction* = 6.94 (.01)
**HPC**	*App*	0.03 (.96)	1.23 (.28)	0.14 (.71)	N/A
	*Aqp4*	0.01 (.93)	0.77 (.39)	7.22 (.01)	N/A
	*Bdnf*	0.01 (.98)	4.15 (.05)	4.56 (.04)	*Injury x Caffeine =* 4.17 (.05)
	*Gfap*	0.63 (.44)	0.98 (.34)	4.14 (.06)	N/A
	*Tau*	9.87 (< .01)	1.52 (.23)	1.82 (.19)	*Sex x Caffeine =* 7.14 (.01)
**HYPO**	*App*	0.03 (.86)	0.73 (.41)	0.83 (.37)	*Sex x Caffeine =* 5.86 (.02)
	*Bmal1*	14.30 (< .01)	17.72 (< .01)	4.45 (.05)	*Injury x Caffeine =* 5.52 (.03)
	*Clock*	1.02 (.32)	7.55 (.01)	0.08 (.78)	*Sex x Injury =* 14.21 (< .01)
	*Cry*	0.39 (.54)	3.50 (.07)	0.37 (.55)	*Three-way interaction* = 4.28 (.05)
	*Orx1*	1.98 (.17)	0.11 (.75)	1.16 (.29)	N/A
	*Orx2*	0.07 (.80)	0.41 (.53)	8.97 (< .01)	*Sex x Caffeine =* 5.04 (.03)
					*Sex x Injury =* 5.01 (.03)
	*Per*	24.45 (< .01)	1.71 (.20)	0.01 (.95)	N/A
	*Tau*	6.92 (.01)	0.22 (.64)	0.19 (.67)	*Sex x Caffeine =* 4.22 (.05)

**Fig 3:**
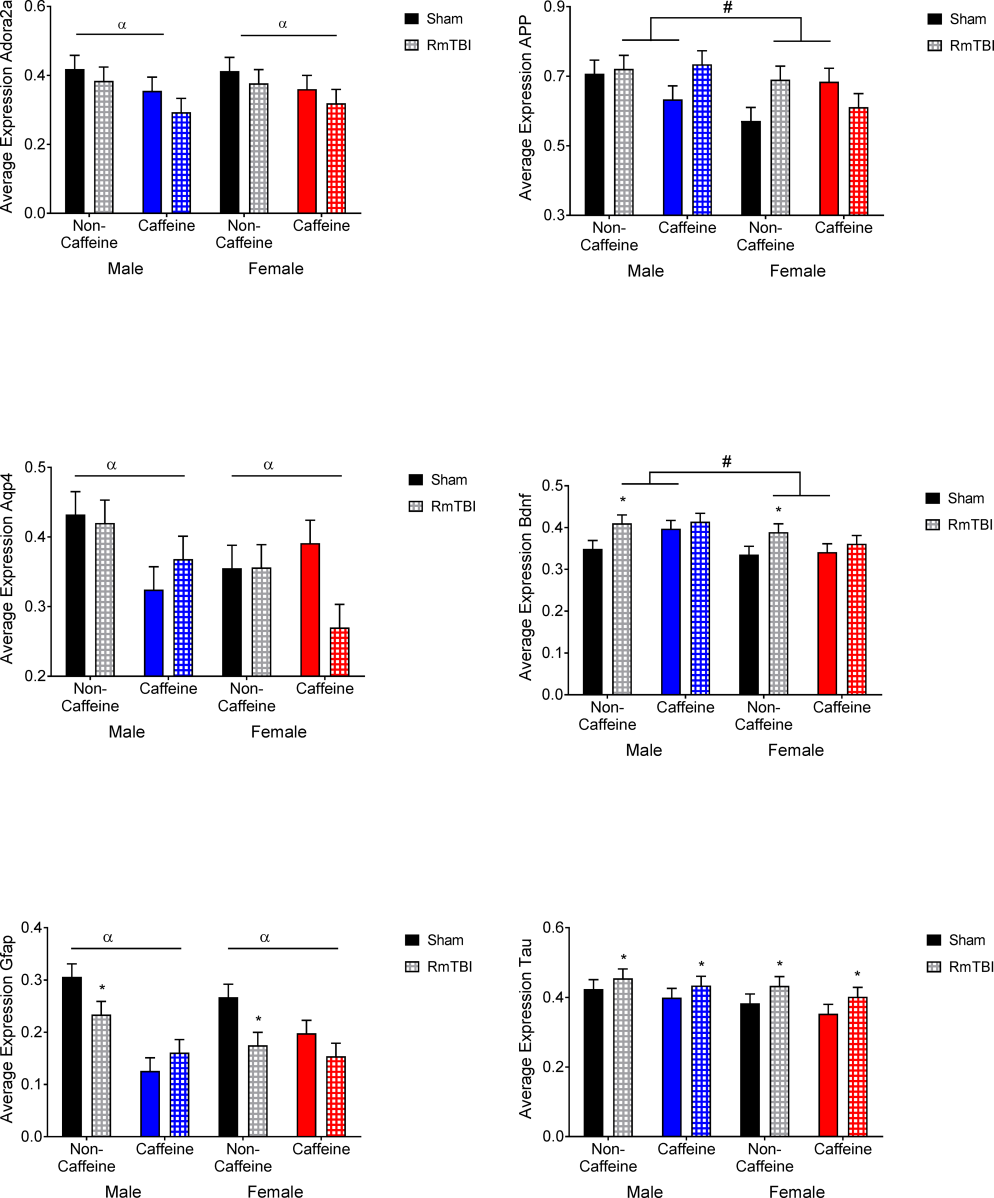
Prefrontal cortex gene expression in male and female sham and RmTBI rats with or without caffeine in the drinking water. Mean differences were considered significant at *p* < .05. (*) indicates a main effect for RmTBI, (α) indicates a main effect for caffeine, and (#) indicates a main effect of sex.

PFC: Graphical representation of the changes in the PFC can be found in Fig 3. The statistical *F* and *p* values are summarized in Table 2. *Adora2A* receptor expression showed a main effect for caffeine with animals in the caffeine groups showing reduced levels. *App* showed a main effect for sex with males having higher levels of expression and a sex by caffeine by injury interaction where *App* increased in the male caffeine RmTBI group, but decreased in the female group. *Aqp4* expression showed main effect for caffeine with the caffeine groups showing decreased levels. *Bdnf* showed a main effect for sex with males showing higher levels of expression and a main effect for injury where male and female non-caffeine groups showed increases in expression following RmTBI. *Gfap* expression showed a main effect for injury where males and females in the non-caffeine groups showed reduced expression following RmTBI, a main effect for caffeine where caffeine groups showed lower expression, a significant sex by caffeine interaction and a significant injury by caffeine interaction. Expression of *Tau* showed a main effect of injury where males and females in both conditions showed increases in *Tau* following RmTBI.

**Fig 4:**
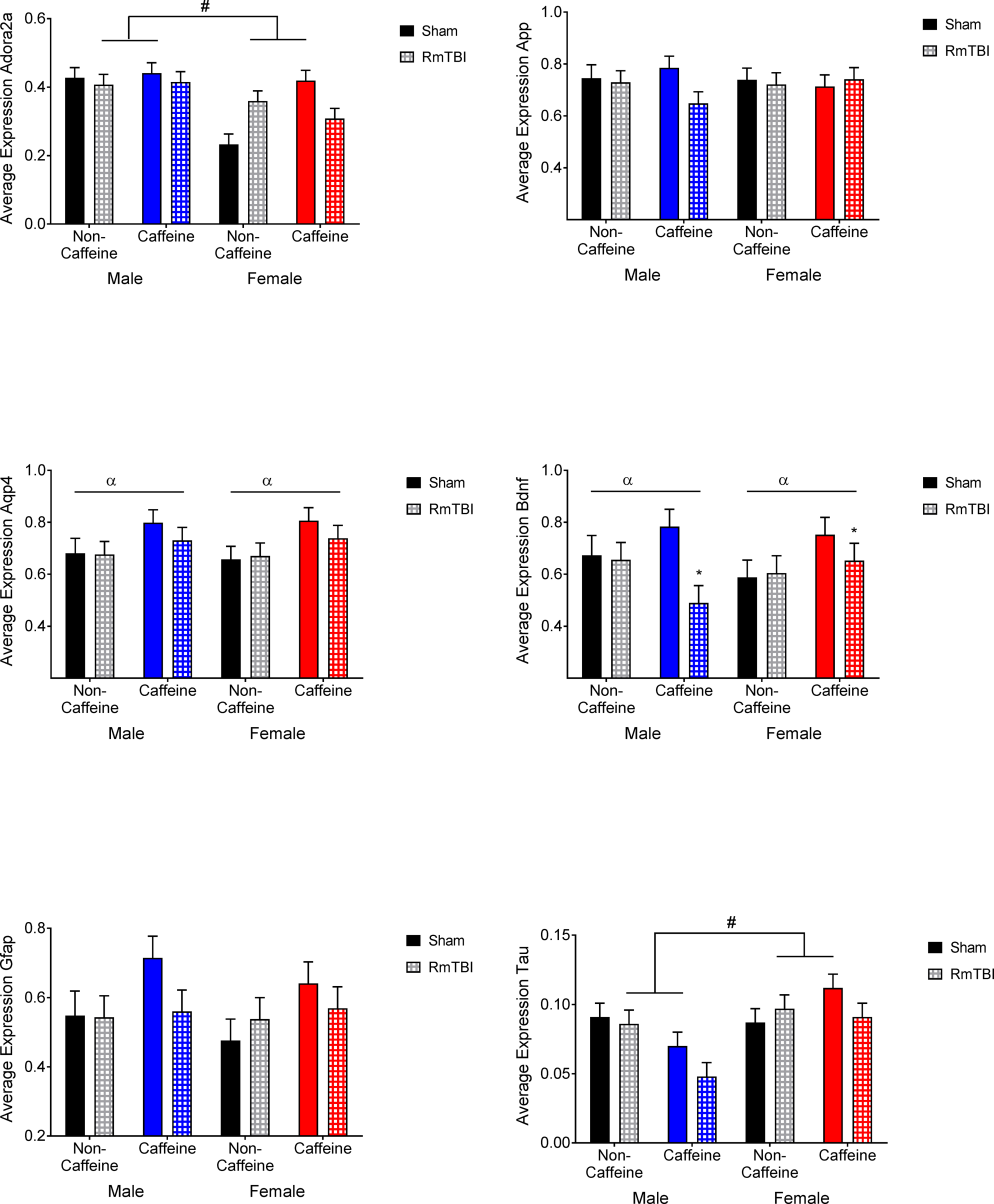
Hippocampal gene expression in male and female sham and RmTBI groups with or without caffeine in the drinking water. Mean differences were considered significant at *p* < .05. (*) indicates a main effect for RmTBI, (α) indicates a main effect for caffeine, and (#) indicates a main effect of sex.

HPC: Graphical representation of the changes in the HPC can be found in Fig 4. The statistical *F* and *p* values are summarized in Table 2. *Adora2A* receptor expression showed a main effect for sex with females showing lower levels, an injury by caffeine interaction and a sex by caffeine by injury interaction. *Aqp4* expression showed a main effect for caffeine where male and female caffeine rats showed increased levels. Expression of *Bdnf* had a main effect from injury with males in females in the caffeine groups showing decreased expression following RmTBI, a main effect for caffeine with sham caffeine animals showing higher levels of expression and an injury by caffeine interaction. Expression of *Tau* showed a significant main effect for sex with females showing higher levels of expression and a sex by caffeine interaction where males in the caffeine group showed decreased expression compared to non-caffeine males, and the female caffeine group showed increased levels compared to non-caffeine females.

**Fig 5:**
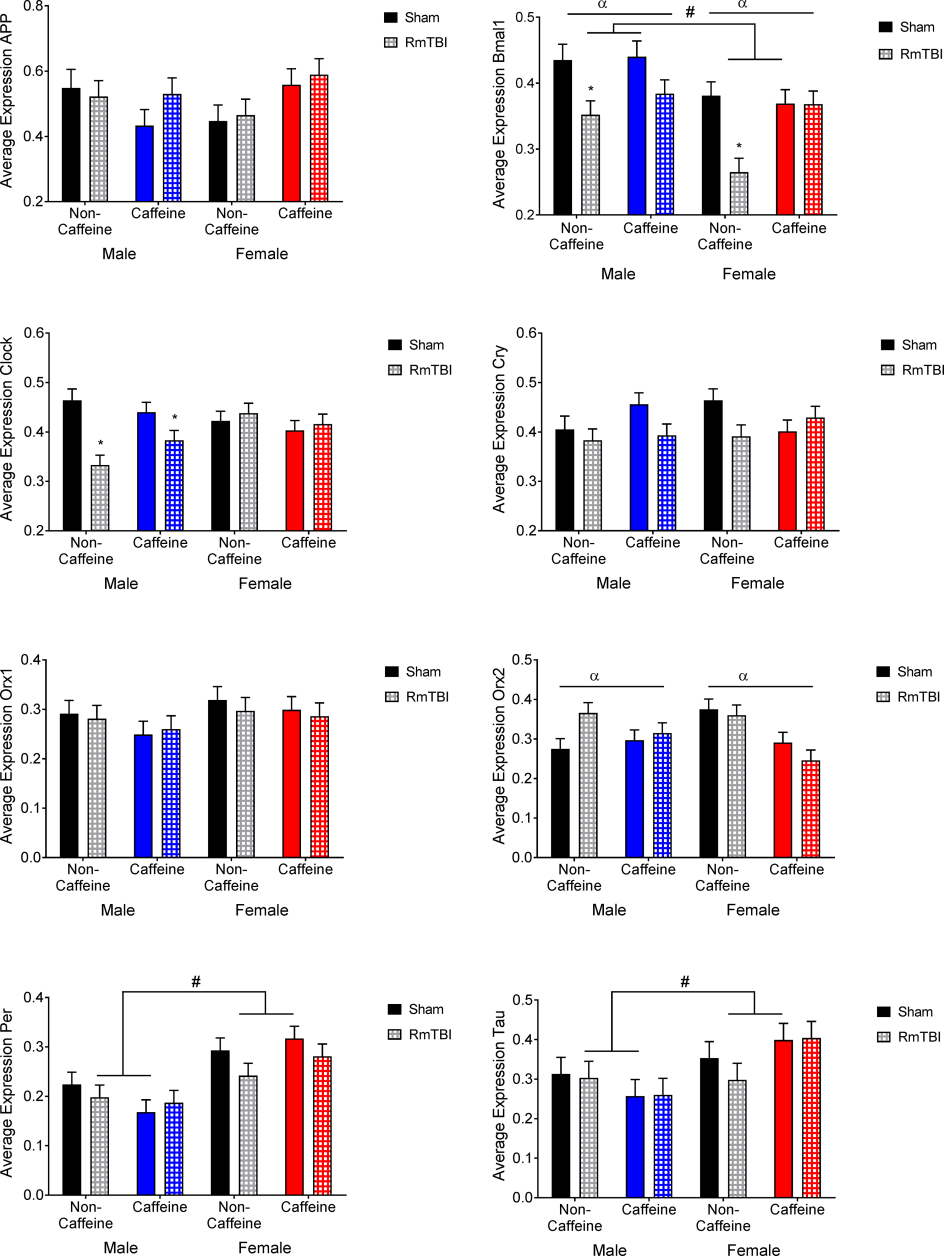
Hypothalamic gene expression in male and female sham and RmTBI groups with or without caffeine in the drinking water. Mean differences were considered significant at *p* < .05. (*) indicates a main effect for RmTBI, (α) indicates a main effect for caffeine, and (#) indicates a main effect of sex.

HYPO: Graphical representation of the changes in the HYPO can be found in Fig 5. The statistical *F* and *p* values are summarized in Table 2. *App* expression showed a sex by caffeine interaction. *Bmal1* showed main effects of sex where males showed higher levels of expression, injury where non-caffeine male and female showed reductions in expression with RmTBI, caffeine, and a significant injury by caffeine interaction. *Clock* expression showed a main effect for injury with males showing reductions in expression following RmTBI in both non-caffeine and caffeine groups and a sex by injury interaction. *Cry* expression showed a sex by injury by caffeine interaction with all groups showing decreases in *Cry* following RmTBI except for the female caffeine group. *Orx2* expression showed a main effect for caffeine with the caffeine groups showing decreased expression, a sex by caffeine interaction and a sex by injury interaction. *Per* expression showed a main effect for sex with females showing higher levels of expression. *Tau* expression showed a main effect of sex with female groups having higher expression in addition to a sex by caffeine interaction where caffeine reduced expression in males but increased it in females.

### Histological analysis

**Fig 6:**
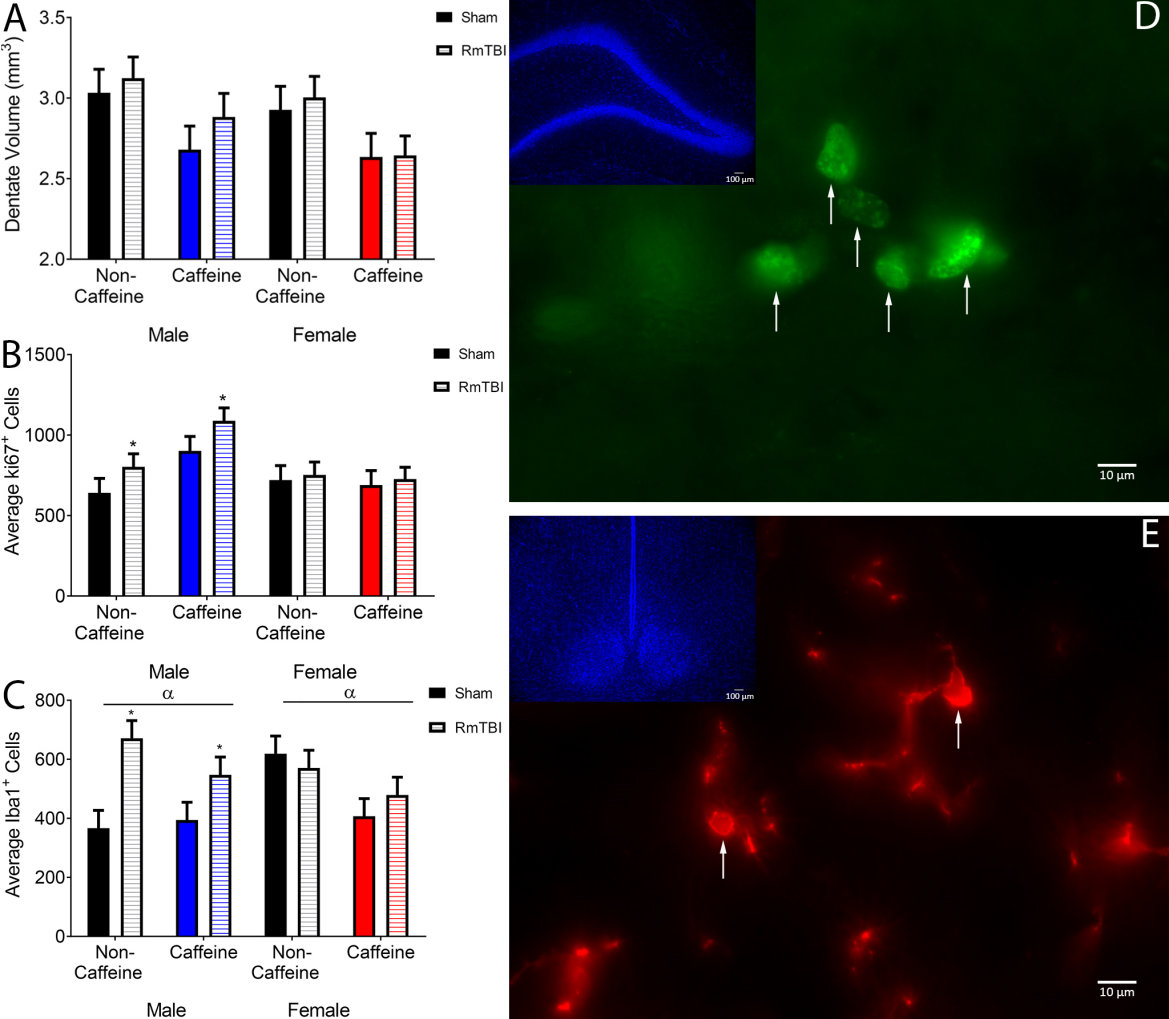
Histological analysis. (A) Displays the mean DG granule cell layer volumes in mm^3^ along with the SEM. Mean differences were considered significant at p< .05. There was a main effect for caffeine, with those rats receiving caffeine showing significantly lower DG granule cell layer volumes. (B) Displays the mean number of cells that were determined to be Ki67 positive in the DG. There was a main effect for RmTBI, with males in both the water and caffeine groups showing significant increases in Ki67 positive cells following RmTBI. (C) Displays the mean number of cells positive for Iba1 in the ventromedial hypothalamus. There was a main effect for RmTBI and caffeine. Males in the non-caffeine and caffeine groups showed significant increases in Iba1 following RmTBI. (D) Is a representative image of Ki67 positive cells in the DG. Arrows highlight cells that were determined to be Ki67 immunoreactive and thus quantified. (E) Is a representative image of Iba1 positive cells in the ventromedial hypothalamus. Arrows highlight cell bodies within the current focal plane that were to be determined to be Iba1 positive and suitable for quantification.

Dentate Gyrus Granule Cell Layer Volume: The volume of the DG granule cell layer was not influenced by RmTBI, but was significantly reduced in male and female rats that consumed caffeine. The three-way ANOVA demonstrated a main effect of caffeine, *F*(1, 35) = 9.97, *p* < .01. None of the other main effects or interactions were significant, *p*’s > .05. See Fig 6A.

Ki67: When compared to females, males exhibited more Ki67 positive cells in the DG. In addition, caffeine exposure increased the number of Ki67 positive cells in males, but reduced the number of Ki67 cells in females. The three-way ANOVA demonstrated a main effect of sex, *F*(1, 35) = 5.24, *p* = .03 and a main effect of caffeine, *F*(1, 35) = 4.23, *p* = .04. There was also a significant sex x caffeine interaction, *F*(1, 35) = 6.41, *p* = .01. See Fig 6B and 6D.

Iba1: Rats exposed to chronic caffeine exhibited a reduction in the number of Iba1 positive cells in the HYPO. Additionally, RmTBI increased the number of Iba1 positive cells in males but not females. The three-way ANOVA demonstrated a main effect of caffeine, *F*(1, 36) = 4.54, *p* = .04, and main effect of injury, *F*(1, 36) = 6.60, *p* = .01. There was also a significant sex x injury interaction, *F*(1, 36) = 5.40, *p* = .02. See Fig 6C and 6E.

## Discussion

### The influence of caffeine on behavioral recovery following RmTBI

Individuals that experience RmTBI often exhibit poorer outcomes [43], but adolescence is a particularly vulnerable window whereby the magnitude and duration of symptoms is inversely related to age [44]. What’s more, caffeine intake has exponentially increased in this population [45, 46]. Although stimulants like caffeine enhance cognitive performance, auditory vigilance, and reaction times [45], they also reduce the brain’s potential for future plasticity [47], and disrupt normal sleep function [48, 49]; two neurological properties needed for recovery from TBI. Therefore, this study examined the effects of adolescent caffeine use on recovery from RmTBI and the susceptibility to post-traumatic symptomology. We found that for males, the negative effects of caffeine and RmTBI were often additive (open field, NCM, and Force Swim), whereby rats that received both had worse outcomes when compared to those that either had caffeine or RmTBI. Conversely, for females, RmTBI and caffeine were equally disruptive. In many of the behavioral measures examined (open field, EPM, and NCM) females with RmTBI were indistinguishable from females exposed to caffeine, with both exhibiting significant impairment when compared to their control counterparts. This suggests that not only are there are sex differences in the way that caffeine interacts with typical brain development; sex also modifies the interaction between caffeine and recovery from RmTBI during this important period of maturation. Sex differences are not surprising, as studies with human populations have demonstrated caffeine to be neuroprotective for Parkinson’s Disease in adult males but not females [50], with follow-up studies in mice indicating that estrogen may actually block the neuroprotective properties of caffeine [51]. While these rats were tested before sexual maturation, sex differences in brain development [52, 53], epigenetic programming [54, 55], and injury response [56–58], are likely modifying caffeine’s effect on the outcomes measured here. Although cognitive effects of TBI and caffeine consumption in youth have not been examined, poor behavioral regulation and performance on executive function tasks like the NCM have been reported in adolescent populations with high caffeine consumption rates [59]. While caffeine’s ability to serve as a therapeutic agent for spinal cord injury, stroke, and neurodegeneration in adults is gaining traction (for review see [60]), the behavioral results from this study suggest that adolescence may represent a unique window of development whereby caffeine exposure is actually detrimental to recovery.

### Caffeine, RmTBI, and changes in gene expression

It has been proposed that RmTBI symptoms result from rapid changes in ionic concentrations and excitatory neurotransmission that increase metabolic demands, change cerebral blood flow, and alter intracranial pressure [2, 61]. Given that these physiological changes would in turn modify gene expression and subsequent protein production, we examined expression changes in 12 genes in the PFC, HPC, and HYPO that we predicted would influence the pathological outcomes associated with early life caffeine exposure and RmTBI. We will begin with a discussion of the PFC. The arousal promoting aspects of caffeine are believed to result from antagonism of adenosine receptors, specifically *Adora2a* [62]. As would therefore be expected, chronic caffeine exposure reduced expression of *Adora2a* in the PFC. Although caffeine and other adenosine receptor antagonists are believed to be neuroprotective [63], caffeine did not reduce markers of neurological damage such as *App*, *Gfap*, or *Tau* following RmTBI in this model. In addition, therapeutic recovery and restoration of cognitive function following TBI has been linked to up-regulation of growth factors such as *Bdnf* [64]. Animals in the control group, but not the caffeine group, exhibited an increase in PFC *Bdnf* expression following the RmTBIs, which may contribute to the lack of recovery identified in these animals.

With respect to the HPC, work in adolescent rats has shown that moderate to high doses of caffeine improved object recognition but also produced anxiogenic effects, decreased density of *Gfap*, and increased *Bdnf* [65]. While we also noted caffeine induced increases in hippocampal *Bdnf* in sham animals, we did not identify significant changes in *Gfap* expression or find improved cognitive abilities. Chronic caffeine has been shown to increase CSF production [66] and *Aqp4* is known to play a role in the reabsorption of fluid in the brain and prevent edema [67], suggesting that the increased expression of *Aqp4* in the HPC of adolescent rats exposed to caffeine may have been a compensatory response to increased CSF flow through the ventricles. Interestingly, and similar to studies examining tau pathology in neurodegenerative diseases, caffeine reduced *Tau* expression in the HPC males, but not females [51, 60]. In female sham animals exposed to caffeine, *Tau* expression actually increased, suggesting that caffeine has adverse effects on the developing brain.

Given that both caffeine and TBI are known to disrupt normal sleep-wake patterns, we also sought to examine changes in expression of circadian clock genes in the hypothalamus. The typical transcriptional feedback loop of gene expression comprising *Clock-Bmal1* and *Per-Cry* constitutes the core circadian clock and generates 24 hr daily rhythms. Caffeine is known to modify circadian rhythms [68, 69] and alter expression patterns of genes such as *Bmal1*, *Clock*, *Cry*, and *Per* in the liver and intestine [70]. Similarly, moderate TBI dysregulates expression of *Bmal1* and *Cry*, and concurrent circadian locomotor activity, suggesting that altered clock gene expression may underlie the sleep disturbances associated with many brain injuries [71]. In this study we found that hypothalamic expression of *Bmal1*, *Clock*, *Cry*, and *Orx2*, were modified by both caffeine exposure and RmTBI. Although we did not measure sleep patterns in these animals, the gene expression results suggest that alterations to circadian rhythms is likely. In addition, with the exception of *Orx1*, all of the genes examined in the HYPO exhibited sex-dependent changes in expression, providing further support to the notion that the pathophysiological response of males and females to caffeine and RmTBI differs significantly.

### RmTBI, caffeine and histological measures

In an effort to understand the pathophysiological changes associated with caffeine exposure and RmTBI in adolescent rats we examined 3 histological measures; DG granule cell layer volume, neuronal proliferation in the DG (Ki67), and microglia activation in the lateral hypothalamus (Iba1). Interestingly, RmTBI did not affect DG granule cell layer volume or the number of Ki67 positive cells in our adolescent rats, but caffeine exposure did. The absence of DG granule cell layer volume reductions following RmTBI was similar to advanced MRI findings in our laboratory that demonstrated no hippocampal volume loss in adolescent rats using this model [72]. These results do however conflict with a recent study in adult rats that found reductions in hippocampal volume following RmTBI [73]; albeit this paradigm may have been more severe, inducing 10 injuries over 10 consecutive days. To our knowledge, there has been little investigation into changes in neuronal proliferation and Ki67 positive cells following mild TBI. However, literature regarding moderate and severe TBI has demonstrated subsequent suppression of proliferating cells (Ki-76 positive) [74, 75]. Although it is possible that the diffuse injuries induced with this model of mild TBI do not disrupt neuronal proliferation in the DG, it is also possible that the lack of RmTBI-induced changes in Ki67 expression are associated with the timing of our sampling, as 20 days post-injury allows for significant recovery.

With respect to caffeine exposure, we found significant decreases in DG granule cell layer volume in both male and female rats. Clinical studies regarding caffeine consumption and hippocampal volume are somewhat inconsistent, with some demonstrating chronic caffeine exposure reduces hippocampal volume [76], and others finding increased hippocampal volume [77]. Animal studies have not examined DG volume, but studies with adult rats have found increased dendritic arborization in the hippocampus following chronic caffeine consumption [78]. In addition, we found that caffeine exposure increased the number of Ki67 positive cells in males, but not females. Previous studies have reported that chronic caffeine consumption reduced BrdU positive cells in the DG in adult rats suggesting reductions in hippocampal neurogenesis [79]. Caffeine has also been shown to be neuroprotective and prevent reductions in Ki67 positive cells in neonatal rats exposed to hyperoxia [80], and in an aged rats modeling Alzheimer’s Disease [34], but there is little evidence to suggest increased Ki67 positive cells following caffeine consumption. It is possible that the differential rates of caffeine clearance and metabolism in adolescent versus adult rats [81] is influencing the neuropathophysiological response to caffeine and in turn generating results that conflict with previous literature (most of which is obtained from much older rats).

Both RmTBI and caffeine exposure modified the number of Iba1 positive cells, an immunohistochemical marker of microglial activation [82], in the ventromedial hypothalamus. As demonstrated in the sex by injury interaction, the number of Iba1^+^ cells were significantly increased in all males that experienced RmTBI, but not in either group of injured females. Given that microglia act as the primary defense mechanism for the brain, scavenging for damage and other pathological changes, RmTBI-induced increases in microglia activation are expected. It is therefore somewhat surprising that females did not exhibit any increases in Iba1 positive cells in response to injury. However, it has also been suggested that caffeine may directly reduce microglia activation by blocking their adenosine receptors [83]. The results for females in this study support this finding, as both sham and RmTBI females chronically exposed to caffeine, exhibited reductions in Iba1 positive cells. The differences identified in Iba1 expression for males and females may reflect fundamental sex differences in injury trajectories and neurological effects of caffeine.

### Conclusions

This study found that chronic caffeine consumption in adolescence altered normal developmental trajectories, as well as recovery from RmTBI. Similar to previous findings in our laboratory [10, 58], many outcomes exhibited sex-dependent responses whereby the sex of the animal modified response to caffeine, RmTBI, and the combination of the two. It is important to note that many studies have demonstrated neuroprotective properties of caffeine, which is contradictory to our findings. We believe that age is a primary contributor to these discrepancies in study results. In contrast to adults, chronic stimulation of the developing brain may not be helpful and may actually have negative consequences. Given the exponential rise in caffeine consumption in adolescents, their increased risk of RmTBI, and the significant amount of brain maturation that is occurring during this time period, these adverse findings warrant further investigation. In addition, future studies should examine sleep wake cycles in these animals, as poor sleep may be contributing to the symptom exacerbation we identified in our caffeine group. Taken together however, these results suggest that caffeine consumption in adolescents at high risk for RmTBI should be monitored, and studies should assess this relationship in clinically relevant populations.
